# Immune-Based Therapeutic Strategies for Acute Myeloid Leukemia

**DOI:** 10.3390/cancers14010105

**Published:** 2021-12-27

**Authors:** Matthias Böhme, Sabine Kayser

**Affiliations:** 1 Medical Clinic and Policlinic I, Hematology and Cellular Therapy, University Hospital Leipzig, 04103 Leipzig, Germany; matthias.boehme@medizin.uni-leipzig.de; 2 NCT Trial Center, National Center of Tumor Diseases, German Cancer Research Center (DKFZ), 69120 Heidelberg, Germany

**Keywords:** acute myeloid leukemia, immunotherapy, bispecific and dual antigen receptor-targeting antibodies, chimeric antigen receptor T-cell therapies, T-cell immune checkpoint inhibitors

## Abstract

**Simple Summary:**

This review summarizes various therapeutic immune approaches representing their targets, the efficacy and toxicity in the treatment of acute myeloid leukemia. In particular, immune checkpoint inhibitors, bispecific T-cell engager antibodies and chimeric antigen receptor-T-cell approaches are highlighted.

**Abstract:**

The development and design of immune-based strategies have become an increasingly important topic during the last few years in acute myeloid leukemia (AML), based on successful immunotherapies in solid cancer. The spectrum ranges from antibody drug conjugates, immune checkpoint inhibitors blocking programmed cell death protein 1 (PD1), cytotoxic T lymphocyte antigen 4 (CTLA4) or T cell immunoglobulin and mucin domain containing-3 (TIM3), to T-cell based monoclonal and bispecific T-cell engager antibodies, chimeric antigen receptor-T-cell (CAR-T) approaches and leukemia vaccines. Currently, there are many substances in development and multiple phase I/II studies are ongoing. These trials will help us to deepen our understanding of the pathogenesis of AML and facilitate the best immunotherapeutic strategy in AML. We discuss here the mode of action of immune-based therapies and provide an overview of the available data.

## 1. Introduction

Acute myeloid leukemia (AML) is characterized by medullary or peripheral presence of immature, undifferentiated blast cells. Per its definition, AML is characterized by ≥20% blast cells in the bone marrow or peripheral blood, and the infiltration of extramedullary tissues is also possible. The accumulation of various somatically acquired genetic changes in hematopoietic progenitor cells that alter normal mechanisms of self-renewal, proliferation, and differentiation leads to its genetic heterogeneity [1]. Currently, the following cytogenetic abnormalities are considered as AML-defining: t(15;17), t(8;21) and inv(16)/t(16;16), irrespective of blast percentage [2].

AML is typically a disease of the elderly with a median age of 72 years at diagnosis and an incidence of 3–4 per 100,000 adults per year. Overall, outcome is poor with a 5-year survival rate of ~40%, which is rapidly declining with increasing age at diagnosis. Outcome is influenced by multiple factors, both patient-dependent, such as pre-existing comorbidities, and disease-dependent, such as cytogenetics, molecular abnormalities and response to initial therapy. Nevertheless, ~50% of younger (≤60 years) and about 80–90% of older patients relapse after achieving their first complete remission (CR). Despite intensive consolidation therapy, the majority of relapsed patients succumb to their disease [3,4].

Based on the recent risk stratification by genetics [5,6], patients can be stratified into three distinct subgroups: favorable, intermediate and adverse risk, of which only the favorable risk group of patients may be cured by chemotherapy alone. For all other patients, only allogeneic hematopoietic stem cell transplant (allo-HSCT) remains a potentially curative treatment option.

Until recently, similar chemotherapeutic approaches were used for the majority of patients [7]. Due to the discovery of new genetic abnormalities in AML, treatment options have expanded over the last few decades and ten agents have recently been approved by the Food and Drug Administration (FDA) in the United States. In Europe, the European Medical Agency (EMA) approved eight new targeted agents as therapeutic approaches in AML [8].

Another promising therapeutic avenue has been opened relying on immune-based approaches.

The principal idea is that leukemic stem and blast cells express aberrant antigens, which differ from the immunophenotype of normal hematopoietic stem cells. Thus, they may represent a potential target of attack for the immune system, which can be directed towards the disease either by antibody drug conjugates (ADC) or T-cell-based strategies.

**Figure 1:**
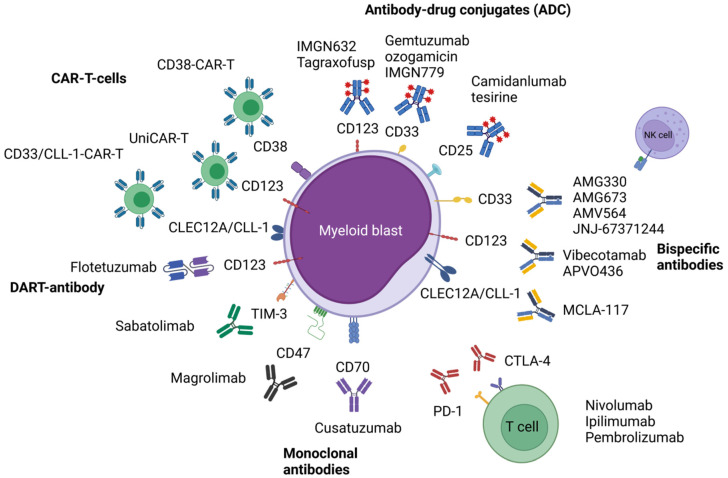
Illustrated overview of the presented immune-based therapeutic approaches in acute myeloid leukemia. Abbreviations: CAR-T, chimeric antigen receptor T-cell; CD, cluster of differentiation; CLEC12A, C-type lectin domain family 12 member A; CLL-1, C-type lectin-like molecule-1; CTLA-4, cytotoxic T-lymphocyte-associated protein 4; PD-1, programmed cell death protein 1; TIM-3, T-cell immunoglobulin and mucin-domain containing-3. Figure 1 was created with BioRender.com (accessed on 14 November 2021).

This review focusses on the available clinical data of immune-based strategies including monoclonal antibodies (mAb) and ADCs, as well as T-cell-based therapeutics, such as bispecific antibodies, immune checkpoint-based approaches and chimeric antigen receptor-T-cells (CAR-T cells). Figure 1 shows an overview of the presented immune-based therapeutic approaches in AML. Most of the drugs/approaches are currently being evaluated in early phase I/II-trials. Thus, clinical data might be preliminary with safety and efficacy evaluations ongoing. Nevertheless, the latest results presented recently on large, international meetings are included. 

## 2. Modalities of Immune-Based Therapeutic Approaches

The administration of mAb may use different pathways to interfere with leukemic stem cells and blast cells. Through recognition of specific antigens, growth signaling may be disturbed and immunological defense pathways activated, for instance complement-mediated cytotoxicity (CDC), as well as antibody-dependent cell-mediated phagocytosis and antibody-dependent cellular cytotoxicity (ADCC). The release of antigens facilitated by such mechanisms leads to presentation by antigen-presenting cells and subsequent activation of CD8+ cytotoxic T-cells, which may directly attack and destroy leukemic cells [9].

However, through clonal evolution, all neoplastic cells have developed some degree of immune evasion, which may be based on different principles. Known examples comprise enhanced signaling of inhibitory immune checkpoints, such as programmed cell death protein 1 (PD1), cytotoxic T lymphocyte antigen 4 (CTLA4) and T cell immunoglobulin and mucin domain containing-3 (TIM3). In addition, inhibitory regulatory T cells (T_reg_) are promoted among others. The disruption of such signals by the use of immune checkpoint inhibitors is a promising approach in AML [9]. 

The upregulation of CD47, which is a “don’t eat me” signal and strongly expressed in solid tumors and myeloid malignancies, results in inhibition of phagocytosis by macrophages and, thus, represents another approach of tumor immune evasion [10,11]. In preclinical models of AML and myelodysplastic syndromes (MDS), it could be demonstrated that the blocking of CD47 enhances antitumor response [12,13] and that the anti-CD47 antibodies stimulate ADCP, promoting priming and memory response of CD8 T cells [14].

Another mode of targeting leukemic cells consists of ADCs, which combine a cytotoxic agent chemically linked to a mAb, thus using a directional way of delivering conventional chemotherapy. By binding to its ligand, the ADC is internalized by the target cell and releases its payload, resulting in cell death of the leukemic cell [9].

A further therapeutic modality consists of bispecific antibodies, which comprise two or more antigen-binding sites in a single antibody construct. One site recognizes a specific antigen on the leukemic cell, whereas the other binds to the effector T cell domain CD3ε, thus allowing for immune cell activation independently of conventional major histocompatibility complex (MHC). The concept of bispecific antibodies allows for different constructs, such as bispecific T cell engagers (BiTEs) or dual-affinity retargeting antibodies (DART^®^) [15]. The latter are described as two variable antigen-specific domains connected to two polypeptide chains, which are in turn linked covalently and non-covalently, improving the stability and crosslinking abilities of similar constructs.

A similar, yet different, therapeutic path lies in the use of CAR-T cells and analogous concepts. Despite their success in the treatment of lymphatic diseases, such as non-Hodgkin lymphoma and acute lymphoblastic leukemia (ALL) [16], current CAR-T approaches had only limited efficacy in AML due to the lack of suitable targets and a hostile immune microenvironment. Therefore, other ideas have been developed, i.e., the use of natural killer cells (CAR-NK), the application of T cell-receptor (TCR)-modified T cells and modular approaches to accommodate for safety concerns, such as the UniCAR system. In addition, vaccinations against leukemic antigens are currently being studied in clinical trials. Table 1 gives an overview of the different immune-based strategies currently being explored in clinical trials.

## 3. Clinical Evaluation of Different Approaches

### 3.1. Antibody Drug Conjugates

One of the first major antigens in the focus of translational research is CD33, also known as Siglec-3 (sialic acid binding Ig-like lectin 3). This transmembrane receptor is overexpressed on leukemic blasts and stem cells and marks myeloid differentiation. Upon activation, it dimerizes and is internalized, rendering it an ideal candidate as a targeted treatment [20].

#### 3.1.1. Gemtuzumab Ozogamicin (GO; Anti-CD33 Monoclonal Antibody)

Currently, gemtuzumab ozogamicin (GO), an anti-CD33 monoclonal antibody, is the only approved therapeutic agent targeting CD33 [20,21]. However, both medical agencies (FDA and EMA) have used different labels, as the FDA indicates the use of GO for both newly diagnosed as well as relapsed or refractory (r/r) CD33-positive AML in adults, whereas the EMA indicates its use only for newly diagnosed AML [17,18,22,23]. After initial approval of GO in 2000, the pharmaceutical company withdrew GO from the market due to an increased mortality rate. GO was eventually re-approved in 2017 after a meta-analysis of 3325 adults demonstrated a survival benefit in patients with favorable- and intermediate-risk cytogenetics [19].

In comparison, early clinical trials of the unconjugated CD33 mAb lintuzumab without an added cytotoxic substance showed no clinical efficacy [24], yet clinical trials using conjugations with short-lived radionuclides such as 225Ac (α-particle decay) are underway (e.g., triple combination of venetoclax, azacitidine and lintuzumab-Ac225 in r/r AML patients (NCT03932318)).

#### 3.1.2. IMGN779 (ADC)

An additional CD33 ADC is IMGN779, which uses a novel DNA-alkylating payload and demonstrated pre-clinical activity in cell lines and xenograft models [25]. A phase I study (NCT02674763) has been completed and demonstrated limited clinical efficacy. Seventy-nine percent of 50 patients, treated with different intravenous administration schedules and doses up to 0.7 mg/kg, showed a decrease in peripheral circulating blasts and 41% demonstrated a >30% reduction in bone marrow blast cells. The most frequently observed adverse event (AE) was febrile neutropenia in 40% of patients.

#### 3.1.3. Camidanlumab Tesirine (ADC, Development Stopped for AML)

The ADC camidanlumab tesirine (also known as ADCT-301), which is directed against CD25 (α-subunit of the interleukin-2 receptor), was evaluated in a phase I trial for patients with r/r AML or ALL (NCT02588092). Camidanlumab tesirine was given intravenously every three weeks, which was later changed to weekly administration. Of the 35 enrolled patients, 16 had post-baseline disease evaluation, of which two showed a CRi. The most common Grade ≥3 treatment-emergent AEs were febrile neutropenia (25.7%), as well as laboratory findings, such as lymphopenia, neutropenia, thrombocytopenia (14.3% each). Pneumonia, increased gamma-glutamyltransferase, and hypophosphatemia occurred in 11.4% each [26]. However, the study was terminated prematurely due to slow recruitment, limited efficacy in this population and early efficacy signals in lymphoma. 

#### 3.1.4. Cusatuzumab (Anti-CD70 Antibody)

Cusatuzumab (anti-CD70 antibody, formerly known as ARGX-110) combined with azacitidine indicates promising results in older patients with newly diagnosed AML of 83% (*n* = 10/12) [27]. Currently, a phase I clinical trial examining the efficacy of the triple combination therapy of cusatuzumab, venetoclax and azacitidine is ongoing (NCT04150887). However, triple combinations of antibody therapy as well as venetoclax + azacitidine are likely to be associated with a high degree of hematological and non-hematological toxicities, such as prolonged cytopenia as well as febrile neutropenia as compared to venetoclax + azacitidine.

#### 3.1.5. IMGN632 (ADC)

The CD123 antibody IMGN632 is conjugated to an alkyl-benzodiazepine and was investigated as a single-agent in 74 patients (7 patients with blastic plasmadendritic cell neoplasm (BPDCN) and 67 patients with AML). The dosage ranged from 0.045 to 0.3 mg/kg IMGN632 per course. Fifty-five percent of the patients with AML showed a reduction in bone marrow blast cells and 20% achieved a CR/CRi. Additionally, 43% of patients with BPDCN achieved a CR/CRi. The most common AEs included diarrhea (30%; all ≤grade 2), febrile neutropenia (27%; all grade 3), nausea (26%; one grade 3), chills (23%; all ≤grade 2), and lung infection (22%; ≥grade 2). The principal treatment-related AEs were infusion-related reactions (16%; four grade 3), which included chills, nausea, diarrhea and tachycardia. However, none required treatment discontinuation [28]. 

Several studies are currently ongoing evaluating IMGN632 as monotherapy in patients with r/r BPDCN and MRD-positive AML or in combination with azacitidine and/or venetoclax (NCT04086264) [29].

#### 3.1.6. Tagraxofusp (ADC)

Tagraxofusp is an intravenously administered CD123-directed cytotoxin. This treatment was approved by the FDA as treatment of patients aged ≥2 years with BPDCN in December 2018 [30], based on a publication showing 90% ORR in treatment-naive patients of whom 45% reached CR. The 2-year overall survival (OS) rate was 52% [31]. Forty-five percent (*n* = 13/29) of the patients could be successfully bridged to allo-HSCT. Additionally, patients with relapsed/refractory BPDCN had an ORR of 67% and median OS of 8.5 months after treatment with tagraxofusp. Two deaths due to capillary leak syndrome occurred [31]. Currently, the triple combination of tagraxofusp, hypomethylating agents and venetoclax is being evaluated in an ongoing phase 1/2 clinical trial in patients with newly diagnosed CD123-positive AML or high-risk MDS (NCT03113643).

#### 3.1.7. Talacotuzumab (ADC)

The CD123 mAb talacotuzumab in a combined therapy with decitabine was halted prematurely in its clinical development after showing an unfavorable benefit/risk ratio and insufficient efficacy in a phase II/III trial (NCT 02472145). No difference in the CR rate could be observed between patients receiving decitabine monotherapy (11% vs. 15%; *p* = 0.44) or the combination therapy of decitabine with 9 mg/kg talacotuzumab. The most common AEs leading to death included sepsis (4.8%), multiple organ dysfunction (5.4%), pneumonia (3.4%), septic shock (3.4%) and sudden death (0.7%). The most common reported infusion-related AEs were chills (16.3%), pyrexia (5.4%), and hypoxia (4.8%) [32].

### 3.2. Bispecific Antibodies (CD3 x AML Antigen)

#### 3.2.1. AMG330 (BiTE)

AMG330, a BiTE anti-CD33 and anti-CD3-antibody, demonstrated potent antibody-mediated cytotoxicity in experimental AML cell lines and xenotransplantation models [33,34,35]. AMG330 is currently being evaluated in an ongoing phase I trial for r/r AML patients (NCT02520427). Due to its short half-life of less than two hours, AMG330 has to be administered as a continuous intravenous infusion with doses up to 720 µg/day. Updated results have been published [36]. So far, 8 of 42 evaluable patients showed responses consisting of three CRs, four CRis and one morphologic leukemia-free state (MLFS). As expected, the most frequent observed AE was cytokine release syndrome (CRS), which occurred in two-thirds of the patients and with grade 3 or higher in 13% of patients. It correlated with both dose level and disease burden.

#### 3.2.2. AMG673 (BiTE)

As stated before, the short half-life of AMG330 renders continuous infusion necessary. AMG673 is a modified CD33/CD3 BiTE with an extended half-life due to fusion of an additional IgG Fc chain, allowing a drastic reduction in infusion times in an ongoing phase I trial (NCT03224819), which is active, but currently not recruiting. Out of 38 patients, treated with doses up to 110 µg, 27 were evaluable. A blast cell reduction ≥50% could be observed in six patients. One patient achieved a CRi and went on to allo-HSCT. Overall, 63% of the patients showed a CRS, which was grade 3 or higher in 18% of these patients. In addition, treatment-emergent SAEs were reported in 68%, with infections (34%) being the most common [37].

#### 3.2.3. AMV564 (BiTE)

Another bispecific CD33/CD3 antibody is AMV564, currently under investigation in a phase I trial for patients with r/r AML (NCT03144245). So far, 36 patients could be enrolled with doses up to 300 µg/day and no grade 3 or higher CRS was observed. Thirty-five patients were evaluable and one CR, one CRi and one PR have been achieved [38].

#### 3.2.4. JNJ-67371244 (BiTE)

The bispecific antibody JNJ-67371244 is being investigated in an ongoing phase I trial (NCT03915379) for patients with r/r AML or MDS. Patients are receiving the antibody either subcutaneously or intravenously and must not be eligible for allo-HSCT. No preliminary data have been published yet.

#### 3.2.5. Flotetuzumab (DART Antibody)

The alpha chain of the interleukin-3 receptor, also known as CD123, is expressed on hematopoietic stem/progenitor cells (HSPCs) and myeloid cells and strongly on leukemic stem cells. Increased CD123 expression is associated with higher risk of relapse [39]. 

Currently, flotetuzumab, a combined CD123- and CD3-DART antibody, was evaluated in a phase I/II trial in 88 patients ≥18 years with r/r AML (NCT02152956) [40], and in patients up to 20 years with relapsed or refractory MDS (NCT04158739). The recommended phase II dose was 500 ng/kg/day by continuous infusion. The dosage was escalated during the first week of treatment. Pre-medication included 10–20 mg i.v. dexamethasone. A CR/CRi rate of 30% was reached and a median OS of 10.2 months (range, 1.9–27.3 months) could be observed, with 6- and 12-month survival rates of 75% and 50%. CRS occurred in almost all patients (grade 3 in 8%). Most cases were transient and reversible and 32% of CRS events were observed in the first week of therapy during step-up dosing. Strategies to mitigate CRS included the early use of tocilizumab as well as lead-in dosing [40]. 

#### 3.2.6. XmAb 14045 (BiTE)

In an ongoing phase I dose-escalation study (NCT02730312) for r/r AML patients, the CD123/CD3 bispecific antibody vibecotamab (also known as XmAb 14045 or SQZ622) is being evaluated. In a preliminary report, 104 AML patients, 1 CML patient and 1 patient with B-ALL with a median of three prior lines of therapy were treated with weekly iv administrations and a dosage ranging from 0.003 µg/kg to 12 µg/kg. In higher dose levels (0.75 µg/kg), response could be achieved with an ORR of 14% (*n* = 7/51, CR/CRi/MLFS) and a fast onset of antileukemic activity. As expected, CRS was the most frequent AE, occurring in 62 of 106 patients (85% grade 1–2, 15% grade ≥ 3). Additional mild to moderate AEs were associated with CRS, such as chills, fever, tachycardia, and hypotension and occurred in 24% of the patients. No myelosuppression requiring dose modification or tumor lysis syndrome occurred [41].

#### 3.2.7. APVO436 (BiTE)

Another bispecific CD123/CD3 antibody is APVO436, which is currently being evaluated in an ongoing phase I trial (NCT03647800). The study recruits r/r AML and MDS patients, not fit for intensive chemotherapy or allo-HSCT. Preliminary results of 34 evaluable patients with r/r AML receiving a weekly infusion of APVO436 at dose levels between 0.3 µg/kg and 60 µg/kg demonstrate antileukemic activity, with two patients achieving a PR, which later deepened to CR; additionally, six patients showed prolonged stable disease (SD). Manageable side effects included CRS in 21.7% of the patients (grade ≥ 3 in 8.7% of patients) [42].

#### 3.2.8. CLEC12A/CLL-1: MCLA-117 (BiTE)

The bispecific mAb MCLA-117 uses another target in combination with CD3, namely CLEC12A (also known as CLL-1, C-type Lectin-like molecule 1) and is currently under investigation in an ongoing phase I trial (NCT03038230). CLEC12A is highly expressed on AML blasts and LSCs, potentially resulting in superior eradication while saving normal hematopoietic stem cells. Patients with r/r AML or very high risk MDS can be included. After an initial ramp-up phase, a weekly infusion of the target dose is being administered. Out of 58 evaluable patients, six patients demonstrated a blast cell reduction ≥50% from baseline, including one patient with MLFS, whereas 36.2% of patients experienced CRS (8.6% with grade 3 or higher). No dose-limiting toxicities (DLTs) have been observed [43].

### 3.3. Immune Checkpoint Inhibitors

#### 3.3.1. PD-L1, PD-1 and CTLA-4 Inhibitors

Immune checkpoint inhibitors have shown only very modest clinical efficacy as single agents in patients with r/r AML [44]. However, treatment with hypomethylating agents (HMAs) has shown to upregulate the surface expression of PD-L1, PD-1, PD-L2 and CTLA-4 [45,46]. Patients with the highest expression tend to have poor survival and the shortest period of response to HMA therapy. Thus, the combination of HMAs and immune checkpoint inhibitors may improve outcome [47].

- Avelumab (PD-L1 Antibody)

The PD-L1 antibody avelumab in combination with azacitidine was recently evaluated in a phase Ib/II study in 19 patients with r/r AML [48]. The most common grade ≥3 treatment-related AEs were neutropenia and anemia in 2 patients each. The clinical benefit was only marginal with an overall CRi rate of 10.5%, and a median OS of 4.8 months. However, PD-L2 expression measured by mass cytometry was significantly higher as compared with PD-L1 on AML blasts from all patients who were analyzed at all time points. These data suggest a novel potential role for PD-L2 as a means of AML immune escape. Another PD-L1 antibody, atezolizumab, was evaluated in combination with immunomodulatory agents in a phase 1 study (NCT02892318).

- Nivolumab (Anti-PD1 Antibody)

Nivolumab as combined therapy with azacitidine was recently evaluated in an open label, single arm phase I/II study in 70 patients with r/r AML [49]. The ORR was 33% (whole cohort, HMA pretreatment allowed) including 15 (22%) patients with CR/CRi, and 58% in HMA-treatment naïve patients. Pre-therapeutic analysis of CD3 and CD8 in bone marrow and peripheral blood by flow cytometry were significantly predictive for outcome and response. Grade 2–4 immune-toxicity-associated AEs, such as skin rash, pneumonitis, infections, nephritis, hypophysitis, increased transaminases as well as colitis occurred in 20–25% of the patients and were in 95% reversible, if steroids were started within 24 h [49]. 

A phase I/II trial (NCT02397720) evaluating azacitidine, nivolumab and ipilimumab is currently recruiting in patients with r/r AML. A CR/CRi rate of 19% (*n* = 7/36) and PR in 3% (*n* = 1) could be observed. Interestingly, three of four patients with extramedullary disease achieved PR/CR with a median duration of 8 months. The mortality rate was very low with 0 and 6% after four and eight weeks, respectively. Eight patients (19%) had grade 3 or higher immune toxicities including rash, pneumonitis, colitis, and pyrexia. No deaths caused by immune toxicity occurred. The response correlates with the extension of a cluster of antigen-experienced CD8+ T cells. One-year OS in r/r AML patients after treatment with azacitindine/nivolumab/ipilimumab was 25%. The median OS of 6–8 months after the combined therapy is comparable to that reported with hypomethylating agents and venetoclax salvage in numerous studies [50].

Future directions include the evaluation of the combined therapy of azacitidine + venetoclax and nivolumab in elderly patients (≥ 65 years) with either newly diagnosed or r/r AML.

- Pembrolizumab (Anti-PD1 Antibody)

For the anti-PD-1 mAb pembrolizumab, several trials aiming to implement the additional therapeutic option at different disease stages are underway. Two randomized phase II studies are evaluating pembrolizumab as first-line treatment for patients not eligible for intensive chemotherapy in combination with azacitidine and venetoclax or with intensive chemotherapy for eligible patients, respectively (NCT04284787, NCT04214249). However, no published data are available yet. Another phase I trial (NCT03969446) investigates the addition of pembrolizumab to decitabine in newly diagnosed AML or high-risk MDS as well as in r/r AML patients. Preliminary results in a small cohort have been reported [51]. Ten high-risk AML patients were treated with pembrolizumab 200 mg every 3 weeks and decitabine 20 mg/m^2^ for a total of 10 days for up to eight cycles. This treatment led to an MRD-negative CR in one, SD and progressive disease in four patients each; one patient did not complete the study. The treatment was well tolerated; no grade 5 AE occurred. Most grade 4 AEs were hematological. Grade 4 sepsis occurred in two patients. Two patients suffered from hypothyroidism and a third patient developed central diabetes insipidus, possibly associated with pembrolizumab [51]. 

In addition, pembrolizumab is under investigation as additional agent when using MRD-triggered treatment in combination with azacitidine (PEMAZA, NCT03769532) in patients harboring a *NPM1*-mutation, who are in hematological CR but MRD-positive.

- Ipilimumab (CTLA-4 inhibitor)

Treatment with the anti-CTLA-4 monoclonal antibody ipilimumab showed clinical activity in relapsed patients with hematological malignancies after allo-HSCT, including 5 of 14 patients with AML, who achieved CR [52]. Grade 3/4 immune-related AEs occurred in 25% of the patients within the trial. A phase 1 trial on the combination of ipilimumab and CD25/Treg-depleted DLI in patients with relapsed myeloid diseases after matched-HCT is currently ongoing (NCT03912064).

#### 3.3.2. T-Cell Immunoglobin and Mucin Domain 3 (TIM-3) Inhibitor

Another potent immune checkpoint is T-cell immunoglobin and mucin domain 3 (TIM-3), which is expressed by LSCs as well as a multitude of immune cells.

- Sabatolimab

Sabatolimab (MBG453) is an antibody-based inhibitor, which is being evaluated in an ongoing phase I trial (NCT03066648) in combination with either azacitidine or decitabine [53]. Fourteen of 34 (41%) evaluable patients with newly diagnosed AML showed a response (three patients with PR, three with CRi, and eight patients with CR, respectively). The most common grade 3 or higher treatment-emergent AEs were thrombocytopenia (45.8%), neutropenia (50%), febrile neutropenia (29.2%), anemia (27.1%), and pneumonia (10.4%). However, only three patients needed to discontinue the therapy. One dose-limiting toxicity occurred (grade 3 ALT elevation); the maximum tolerated dose was not reached with either combination [53]. 

Currently, trials investigating the combination therapy with venetoclax are enrolling patients (NCT04150029, NCT04623216).

#### 3.3.3. Macrophage-Based Inhibitor

- Magrolimab

The antibody magrolimab, which targets CD47 as a macrophage immune checkpoint inhibitor, is evaluated in several early clinical trial studies in AML. A phase I trial of magrolimab with azacitidine led to an overall response rate (ORR) of 91% in patients with MDS and a CR rate of 42%. In addition, high response rates were observed in *TP53-*mutated MDS patients (NCT03248479). Of note, AML patients with a *TP53* mutation (*n* = 12) showed a CR/CRi rate of 75%. With a median follow-up of 8.8 months, the median duration of response or OS was not met. The therapy was well tolerated, and no treatment-related febrile neutropenia occurred. Common treatment-related AEs were anemia (44%), fatigue (18%), infusion reaction (18%), neutropenia (8%) and thrombocytopenia (5%). In addition, no patient discontinued due to an AE. The mean decline in hemoglobin levels with the first dose of magrolimab + azacitidine was only 0.4 g/dL. Fifty-eight percent of the patients became red blood cell transfusion independent [54]. 

Altogether, these promising results demonstrate the clinical applicability of macrophage checkpoint blockade. Currently, these observations are being further elucidated in a randomized, double-blind phase III trial for untreated high-risk MDS patients (ENHANCE, NCT04313881) [55]. 

### 3.4. CAR-T Cell Approaches

The principle of chimeric antigen receptor T-cells (CAR T-cells) has had great success in the field of lymphatic diseases, with EMA and FDA approved CAR T-cells for the treatment of multiple myeloma, mantle cell lymphoma and relapsed or refractory diffuse large B-cell lymphoma, respectively. It applies the method of transduction of patient-derived T-cells using viral vectors, thus leading to the expression of genetically modified T-cell receptors and independent tumor-specific antigen recognition, regardless of the presence of co-stimulatory molecules. However, translating this achievement to the field of myeloid malignancies has proven to be difficult as potential target antigens are rare and the specific immune microenvironment of AML is hostile towards immunogenic attack [56]. Moreover, most potential targets are also being expressed by normal hematopoietic stem cells, increasing potential off-target effects and harming the recovery of normal bone marrow. Various approaches are currently under investigation to circumvent these obstacles, as discussed below.

- CD33-CLL-1-CAR-T

Using a unique dual specific approach, an ongoing phase I, first-in-human trial utilized CD33-CLL-1 CAR T-cells in nine patients with r/r AML (NCT03795779) [57]. Four weeks after CAR T-cell infusion, seven patients reached MRD-negativity using flow cytometry, of which six moved on to subsequent allo-HSCT, thus indicating that this might be a strategy to overcome some of the limitations of CAR T-cell therapy. As expected, CRS occurred in almost all patients (*n* = 8; 3 grade Ι, 3 grade ΙΙ, and 2 grade ΙΙΙ) and neurotoxicity in 4 patients (1 grade Ι and 3 grade ΙΙΙ). Pancytopenia grade IV occurred in all patients. Sepsis and pneumonia occurred in three patients each and fungal infection in two patients. All AEs were resolved after treatment. Early intervention with steroids reduced CRS and neurotoxicity [57].

- CD33-CAR-T

The approach of CAR-T cells targeting CD33 is also under examination as a potential immune-based therapy. Currently, a phase I/II trial using a CD33-CAR-T cell is being evaluated in children and young adults with r/r AML (NCT03971799). The construct, using linutuzumab-CD28/CD3ζ, has shown robust in vitro and in vivo activity against cell lines and patient-derived xenograft models [58].

- CAR-T-38

The antigen CD38, expressed on mainly leukocyte subtypes and well known as suitable target for the therapy of multiple myeloma, is also a target for CAR T-cells in r/r AML. Currently, this approach is being evaluated in a phase I/II trial (NCT04351022). Currently, results on six relapsed AML patients after allo-HSCT are available [59]. All patients exhibited CD38 in more than 90% on their blast cells. Four weeks after the CAR-T-38 cells infusion, four of six (66.7%) patients achieved CR or CRi. However, the median duration of CR or CRi was only 6.4 months (range 3.9–8.7 months) and the cumulative relapse rate at 6 months was 50%. Median OS and leukemia-free survival were 7.9 and 6.4 months, respectively. Thus, further approaches are needed to maintain CR/CRi after CAR-T cell approaches. Regarding toxicity, grade I-II CRS occurred in five patients and grade III hepatotoxicity in one patient. All AEs were transient and clinically manageable. Pancytopenia was present in all patients before CAR-T-38 infusion, and neutropenia persisted during CAR-T-38 therapy. The median duration of neutropenia was 22 (range, 7–35) days. The median duration of platelet recovery was 25 (range, 18–123) days. No neurological toxicities or severe infections occurred [59].

- UniCAR

A modification on the CAR-T cell approach is the universal CAR-T platform (UniCAR), in which a universal adapter molecule with a short half-life is being continuously infused intravenously. This allows for quick cessation of CAR-T side effects such as CRS or immune effector cell-associated neurotoxicity syndrome (ICANS) and is currently being explored in a phase I trial (NCT04230265) [60,61]. Very recently, the results of three patients that were dosed and had completed treatment within the UniCAR trial were reported [62]. All patients showed a response (one PR and two CRi). AML was still under control 100 days after UniCAR administration in one patient, whereas another patient relapsed after one month. So far, no DLTs were observed. In two patients, grade 1 CRS occurred, which were manageable within two days with antipyretics [62]. 

Another approach is to use T-cell receptor (TCR)-modified T-cells, which do not express a CAR, while also targeting a specific antigen. One such antigen is the preferentially expressed antigen of melanoma (PRAME), which has been shown to be a potential target for AML therapy in concurrence with specific human leukocyte antigen (HLA) restrictions. A phase I/II trial (NCT03503968) using such T-cells is currently underway.

- CAR natural killer cells

Finally, an alternative approach is to exploit and take advantage of the potential of natural killer cells (NK-cells). An active, recruiting phase I trial (NCT04623944) uses allogeneic, genetically engineered CAR NK-cells (NKX101), which have been modified to express several co-stimulatory and signaling receptors as well as membrane bound interleukin-15 used as an autocrine growth factor [63]. Preclinical studies have shown anti-leukemic activity in xenograft models.

### 3.5. Vaccination Approaches

Another strategy to combat AML presented here is the use of vaccination-based ideas in order to harness the immune systems’ ability to recognize leukemia-specific antigens through common pathways mediated by dendritic cells and T lymphocytes, among others. The most challenging issue for tumor vaccinations remains antigen selection. A recently published trial (NCT03697707) investigated the use of two different vaccination regimes using allogeneic dendritic cells in 12 advanced-stage elderly AML patients who were in CR, but MRD-positive. The study was based on a 3 + 3 dose escalation design. Patients received four biweekly intradermal DCP-001 injections at different dose levels (10, 25, and 50 million cells DCP-001). The treatment was well tolerated. SAEs occurred in six patients, with a possible relationship to study treatment in one (diabetes insipidus). A median OS of 36 months (range, 7 to 63 months) was observed in patients with no circulating blasts in comparison to patients with circulating blasts, who died within 6 months. Maintained T cell levels and multi-functional immune responses were associated with long-term survival [64].

## 4. Conclusions and Future Challenges

Immune-based therapeutic approaches are an important asset in the fight against AML. Currently, there are many substances in development. However, most of the clinical data are mainly available in meeting presentations, since these trials are still in progress. Nevertheless, these preliminary data suggest an acceptable safety profile with promising efficacy. As expected, CRS is one of the most frequently observed AE, whereas neurotoxicity does not seem to be common. Currently, the only approved immune-based therapies are GO for AML and tagraxofusp for BPDCN. Further agents, used as monotherapy or in combination with chemotherapy or venetoclax + HMA, might provide long-term disease control including MRD negativity. However, more studies are needed to evaluate the impact on patient outcomes. 

## Figures and Tables

**Table 1:** Overview of the presented immune-based therapeutic approaches in acute myeloid leukemia currently under clinical investigation.

Target	Drug Name	Drug Type	Therapy	Indication	Developmental Stage	Available Results	NCT Number/ Reference
CD33	Gemtuzumab ozogamicin	ADC	Intensive chemotherapy	de novo AML	EMA and FDA approved therapy	Prolonged EFS and OS	[17,18,19]
	AMG330	BiTE	Monotherapy	r/r AML	Phase I	CR/CRi/MLFS	02520427
	AMG673	BiTE	Monotherapy	r/r AML	Phase I	CRi	03224819
	AMV564	BiTE	Monotherapy	r/r AML	Phase I	CR/CRi/PR	03144245
	IMGN779	ADC	Monotherapy	r/r AML	Phase I	Blast reduction	02674763
	JNJ-67371244	BiTE	Monotherapy	r/r AML	Phase I	N/A	03915379
	CAR T-cells	CAR T-cells	Monotherapy	r/r AML	Phase I/II	N/A	03971799
CD25	Camidanlumab tesirine	ADC	Monotherapy	r/r AML	Phase I, development stopped for AML	CRi	02588092
CD47	Magrolimab	mAb	Azacitidine	MDS/AML	Phase I; randomized, double-blind phase III trial for untreated high-risk MDS patients (ENHANCE, NCT04313881)	Improved CR/CRi	03248479 04313881
CD70	Cusatuzumab	mAb	Azacitidine/ Venetoclax; Azacitidine; Monotherapy	de novo AML, r/r AML	Phase I	Improved CR/CRi	04150887
CD123	Flotetuzumab	DART	Monotherapy	r/r AML	Phase I/II	CR/CRi	02152956
	IMGN632	ADC	Azacitidine/ Venetoclax	r/r AML	Phase I/II	CR/CRi	04086264
	Vibecotamab	BiTE	Monotherapy	r/r AML	Phase I	CR/CRi/MLFS	02730312
	APVO436	BiTE	Monotherapy	r/r AML	Phase I	PR	03647800
	Tagraxofusp	ADC	Azacitidine/ Venetoclax	r/r AML, BPDCN	Phase I; tagraxofusp as monotherapy: approved for BPDCN	N/A	03113643
	Talacotuzumab	mAb	Decitabine	de novo AML	Phase II/III, halted prematurely in its clinical development	No improvement	02472145
	UniCAR	CAR T-cells	Monotherapy	r/r AML, MDS, BPDCN	Phase I	N/A	04230265
CLEC12A/ CLL-1	MCLA-117	BiTE	Monotherapy	r/r AML	Phase I	MLFS, blast reduction	03038230
PD-1/ CTLA-4	Nivolumab	mAb	Azacitidine, Ipilimumab	de novo AML, r/r AML	Phase II	CR/CRi	02397720
	Ipilimumab	mAb	Azacitidine, Nivolumab	de novo AML, r/r AML	Phase II	CR/CRi	02397720
	Pembrolizumab	mAb	Azacitidine	MRD+ AML in CR	Phase II	N/A	03769532
			Azacitidine/ Venetoclax; Intensive chemotherapy	de novo AML	Phase II Phase II	N/A	04284787 04214249
			Decitabine	de novo AML	Phase I	CR, SD	03969446
TIM-3	Sabatolimab	mAb	Decitabine or azacitidine	de novo AML	Phase I	CR/CRi	03066648
			Azacitidine/ Venetoclax	de novo AML	Phase II Phase I/II	N/A	04150029 04623216
CD33/CLL-1	CAR T-cells	CAR T-cells	N/A	r/r AML	Phase I	MRD negative, CR	03795779
CD38	CAR T-cells	CAR T-cells	N/A	r/r AML	Phase I/II	N/A	04351022
PRAME	TCR-modified T-cells	TCR-modified T-cells	N/A	r/r AML, MDS	Phase I/II	N/A	03503968
N/A	CAR NK-cells	CAR NK-cells	N/A	r/r AML	Phase I	N/A	04623944

Abbreviations: ADC, antibody drug conjugates; AML, acute myeloid leukemia; BiTE, bispecific T cell engagers; BPDCN, blastic plasmadendritic cell neoplasm; CAR, chimeric antigen receptor; CD, cluster of differentiation; CLEC12A, C-type lectin domain family 12 member A; CLL-1, C-type lectin-like molecule-1; CTLA-4, cytotoxic T-lymphocyte-associated protein 4; CR, complete remission; CRi, complete remission with incomplete hematological recovery; DART, dual-affinity retargeting antibody; EFS, event-free survival; EMA, European Medical Agency; FDA, Food and Drug Administration; mAB, monoclonal antibody; MRD, measurable residual disease; MDS, myelodysplastic syndrome; MLFS, morphologic leukemia free state; N/A, not applicable; OS, overall survival; PD-1, programmed cell death protein 1; PR, partial remission; r/r, relapse/refractory; PRAME, preferentially expressed antigen of melanoma; SD, stable disease; TCR, T cell-receptor; TIM-3, T-cell immunoglobulin and mucin-domain containing-3.
